# Identification of new heading date determinants in wheat 5B chromosome

**DOI:** 10.1186/s12870-015-0688-x

**Published:** 2016-01-27

**Authors:** Antonina A. Kiseleva, Andrey B. Shcherban, Irina N. Leonova, Zeev Frenkel, Elena A. Salina

**Affiliations:** The Federal Research Center “Institute of Cytology and Genetics of Siberian Branch of the Russian Academy of Sciences”, Prospekt Lavrentyeva 10, Novosibirsk, 630090 Russian Federation; Institute of Evolution, University of Haifa, Mount Carmel, Haifa, 31905 Israel

**Keywords:** Heading date, *VRN-1*, 5B chromosome, Genetic mapping, SNP, QTL, Tetraploid, *Triticum*

## Abstract

**Background:**

Variability of heading date may assist in wheat adaptation to local environments. Thereafter, discovery of new heading date determinants is important for cereal improvement. In this study we used common wheat cultivar Chinese Spring (CS) and the substitution line of CS with 5B chromosome from *T. dicoccoides* (CS-5Bdic), different in their heading date by two weeks, to detect determinants of heading date on 5B chromosome.

**Results:**

The possible influence of the *VRN-B1* gene, the most powerful regulator of flowering, located on 5B chromosome, to differences in heading time between CS and CS-5Bdic was studied. The sequencing of this gene from CS-5Bdic showed that an insertion of a nucleotide triplet produced an additional amino acid in the corresponding protein. No changes in the transcription levels of each homoeologous *VRN-1* loci were found in CS-5Bdic by comparison with CS. To ascertain the loci determining heading date difference, a set of 116 recombinant inbred 5В chromosomal lines as a result of hybridization of CS with CS-5Bdic were developed and their heading dates were estimated. Using the Illumina Infinium 15 k Wheat platform, 379 5B-specific polymorphic markers were detected and a genetic map with 82 skeletal markers was constructed. Phenotype (heading date) – genotype association analysis revealed seventy eight markers in pericentromeric region of 5B chromosome significantly associated with heading date variation. Based on this estimation and synteny with model crop genomes we identified the three best candidate genes: *WRKY*, *ERF/AP2* and *FHY3/FAR1*.

**Conclusions:**

We supposed that the difference in activity of *WRKY*, *ERF/AP2* and/or *FHY3/FAR1* transcription factors between CS and CS-5Bdic to be a probable reason for the observed difference in heading dates. Data obtained in this study provide a good basis for the subsequent investigation of heading time pathways in wheat.

**Electronic supplementary material:**

The online version of this article (doi:10.1186/s12870-015-0688-x) contains supplementary material, which is available to authorized users.

## Background

One of the most important traits in cereal crops is heading date [1]. The transition from vegetative to reproductive growth is a critical developmental stage and a major adaptive trait in both crop and wild cereal species [2]. Wheat cultivars with appropriate heading time to the target environment and life cycle duration will help maximize yield potential in any environment [3, 4]. Heading time in wheat is determined by three major genetic factors: vernalization requirement, photoperiod sensitivity and narrow-sense earliness [5, 6]. The wheat group of chromosome 5 is known to carry genes affecting heading time, such as *VRN-1* [7–9], *PHY-C* [10–12]. For example, *VRN-1* gene encoding a MADS-box transcription factor has been reported to be the key regulator determining vernalization requirement and heading date [13–16]. Estimation of *VRN-1* transcripts levels showed that their concentration must reach a threshold in order to trigger the transition from vegetative to reproductive development [17]. In vernalization-requiring (winter) cereal plants, *VRN-1* is expressed at low levels and is induced by vernalization with the level of expression being dependent on the length of cold exposure [18–21]. Changes in the growth habit of winter forms to spring ones which do not require vernalization, occurred due to dominant mutations in regulatory regions (promoter or intron 1) of *VRN-1*. Using near-isogenic lines, the structural variation within the first intron of *VRN-B1* was shown to be responsible for high differences between the lines in the level of *VRN-B1* transcription and flowering date [22].

Some other loci influencing heading date were shown to be located on 5B chromosome [9, 23–26]. These loci were identified using QTL analysis based on genetic linkage maps. Genetic maps are an important tool for genomic studies, marker-assisted selection, QTL analysis and map-based cloning, physical mapping and evolutionary genomics [27, 28]. There are some maps for *T. durum* x *T. dicoccoides* based on SSR, RFLP, AFLP, RAPD and DArT markers [29]. These markers are not easily amenable to high-throughput genotyping [30]. In contrast to those types of molecular markers, SNPs are ideally suited for the construction of high-resolution genetic maps and discovery of marker–trait associations [31, 32].

In our research we used cv. Chinese Spring (CS, haplotype *vrn-A1vrn-B1Vrn-D1*) and CS-5Bdic, line of CS with chromosome 5B substituted from *T. dicoccoides* to detect determinants of heading date on 5B chromosome. CS was earlier than CS-5Bdic by 15 days. We examined (i) whether the difference in heading date between these lines can be sufficiently explained by the difference in transcription of *VRN-1*, or (ii) it can be explained by the difference in some other loci (QTLs) mapped to 5B. To identify the loci determining difference in heading time we developed a mapping population of recombinant inbred chromosomal lines (RICLs) originated from the cross of CS with CS-5Bdic. The mapping population was genotyped using the Illumina Infinium 15 k Wheat platform. QTL analysis allowed us to identify loci covering 78 SNP markers in the pericentromeric region of 5B chromosome significantly (*p*-value = 0.001) associated with heading date variation. Based on synteny with model crop genomes we identified the three best candidate genes: *WRKY*, *ERF/AP2* and *FHY3/FAR1*.

## Methods

### Plant materials

Chinese Spring (CS), the substitution line of CS with 5B chromosome from *T. dicoccoides* (CS-5Bdic) and F1 seeds of CS x CS-5Bdic were kindly provided by Prof. B. S. Gill (Kansas State University, USA) and J. D. Faris (USDA-ARS, Fargo, USA). A population of 116 recombinant inbred chromosomal lines (RICLs) was developed by 7 generations of self pollination. These lines are expected to contain regions of *T. dicoccoides* 5B chromosome introgressioned into 5B chromosome of CS.

### DNA extraction and purification

DNA was extracted using a sodium bisulfite protocol. Fresh leaf tissue (100 – 150 mg) with 200 μl of warm (60 °C) extraction buffer (0.1 M Tris, 0.5 M NaCl, 0.05 M EDTA, 0.38 % NaHSO_3_, 1.25 % SDS) was homogenized using MP FastPrep-24. Then 500 ml of warm extraction buffer (60 °C) were added, mixed, and incubated for 30 min at 60°. 700 μl of chloroform/isoamyl alcohol (24:1 vol:vol) was added. After centrifugation (15 min, 12000 rpm, Eppendorf 5415 R), the supernatant was collected in new tube and 1.4 ml of 96 % cold (−20 °C) ethyl alcohol was added and mixed gently. After centrifugation (15 min, 12000 rpm) supernatant was removed and DNA pellets were washed in 70 % ethanol, dried and resuspended in 50 μl of TE buffer. DNA purification for SNP genotyping was performed using “Bio-Silica kit for DNA purification from reaction mixtures” according to the manufacturer’s protocol. DNA concentration measurement was performed with NanoDrop M2000 (Thermo Scientific).

### Isolation and analysis of *Vrn-B1* sequences

For isolation of the promoter with adjacent exon 1 of *Vrn-B1* we used primers P1 (5'-TACCCCTGCTACCAGTGCCT-3') and Int1R (5'-GCAGGAAATCGAAATCGAAG-3') reported in [33] and [34], respectively. We isolated the most critical part of the first intron of *Vrn-B1* using primer pair Ex1/C/F // Intr1/B/R4 reported in [35]. The region between exon 2 and exon 8 (ex2-ex8) was split into two subregions and amplified separately as described in [22]. Amplified DNA fragments were recovered from 1 % agarose gels, purified using a QIAGEN MinElute Gel Extraction Kit (QIAGEN, Germany) and directly sequenced using an ABI PRISM Dye Terminator Cycle Sequencing ready reaction kit (Perkin Elmer Cetus, USA) and corresponding specific primers. Sequencing of each fragment was performed in both directions using an ABI PRISM 310 Genetic Analyzer (Perkin Elmer Cetus). The reported nucleotide sequences of *Vrn-B1* alleles from cv. Chinese Spring and substitution line CS-5Bdic were deposited in the EMBL, GenBank, and DDBJ nucleotide sequence databases under accession numbers [GenBank:KT750252] and [GenBank:KT750253], respectively.

### RNA extraction and reverse transcription

Growth conditions of plants used in the current study, RNA extraction and reverse transcription were performed as described in Shcherban et al. [22]. The plants were grown in a greenhouse (20 °C-25 °C) under 12 h of light per day without vernalization. Apices of the leaves of individual plants were cut and pooled at different developmental stages, defined by the number of completely emerged leaves: third (3rd), fourth (4th) and fifth (5th) leaf stages. We used leaf apices of 5 plants per replicate. Two replicates were performed at each developmental stage.

RNA was extracted using RNeasy Plant Mini RNA Extraction Kit (QIAGEN, Germany) from the leaf apices of plants on the different stages before heading: three- leaf, four- leaf and five leaf stages. The RNA samples were checked for genomic DNA contaminations by PCR using primers specific to the ubiquitin gene (UBC) [36]. Single-stranded cDNA was synthesised from 1 μg of total RNA using a (dT)_15_ primer and the QIAGEN Omniscript Reverse Transcription kit in a 20 μl reaction mixture.

### Semi- quantitative RT-PCR

We used specific primers to the *Vrn-A1*, *Vrn-B1* and *Vrn-D1* coding sequences, developed by Shcherban et al. [22]. The PCR mixtures (20 μl) contained 50 ng of cDNA, 0.25 mM each of primer, 0.2 mM dNTPs, 1x reaction buffer (67 mM Tris HCl pH 8.8; 1.8 mM MgCl_2_; 18 mM (NH_4_)_2_SO_4_; 0.01 % Tween 20) and 1 unit of *Taq* DNA polymerase. Amplification was performed using Touchdown PCR (13 cycles of 94 °C for 30 s, 65 °C (−0.5 °C/cycle) for 30 s, 72 °C for 45 s and 24 cycles of 94 °C for 30 s, 60 °C for 30 s, 72 °C for 45 s). PCR products were separated by 1.5 % agarose gel electrophoresis.

### Phenotyping of population

Phenotypic analysis of lines was conducted in the greenhouse under controlled conditions. If vernalization requirement had to be satisfied, the sprouted seeds were vernalized for 30 days at +3 °C in the dark. Evaluation of heading date was calculated as duration of the period from sprouting to heading. The date of heading was subsequently recorded for all plants as the date when 1/3 of spike emerged from the flag leaf. Days to heading were recorded as the number of days from the sprouting to the date of heading. The average value of heading date for every line was calculated for 10 plants.

### SNP genotyping

Using 116 RICLs, parental lines, DT5BL and N5BT5D SNP genotyping was performed using the Illumina Infinium 15 k Wheat platform by TraitGenetics GmbH [37]. Total amount of analyzed loci was 13007. DT5BL and N5BT5D were included in this set for the further localization of markers to short (5BS) or long (5BL) arm of 5B chromosome.

### Screening of RICLs with *Vrn-B1* specific primers

For screening the recombinant inbred lines, the specific primers were used to distinguish *Vrn-B1* alleles from *T. aestivum*, cv. Chinese Spring (CS allele), and CS-5Bdic line (allele of *T. dicoccoides*)*.* Sequences of forward primers dicCSintr5 (5'-CCTTGCATACCTGAACCG-3') and CSintr5 (5'-ACCTTGCATACCTGAACCA-3') were designed in such a way that the last position at the 3′ end matches the SNP in the intron 5 of *Vrn-B1*. These primers were used in combination with common reverse primer Ex8/7 (5'-GCCCTTCAGCCGTTGATGGGCTA-3'). Amplification was performed using the Touchdown protocol with decreasing annealing temperature at 0.5 °C/cycle from 62 °C to 56 °C. Primers pair CSintr5 // Ex8/7 gave a product of about 700 bp in the case of CS allele, whereas no products were obtained in the case of the *T. dicoccoides* allele. Conversely, amplification with pair dicCSintr5 // Ex8/7 gave a 700 bp product in the case of the *T. dicoccoides* allele. The approach allowed us to detect heterozygous alleles.

### Genetic linkage map construction

The genetic linkage map of 5B chromosome was constructed using software MultiPoint version «UltraDense» [38]. Markers with more than 8 missing data-points and markers with large segregation distortion (*χ*^2^ > 21) were removed. Coefficients of priority were 0.9 for missing data and 0.1 for segregation since we expected only one linkage group. The minimum size of group of co-segregating markers was 2. Co-segregating markers are tightly linked markers located in the same position. Ordering of skeletal markers was performed using guided evolutionary strategy (GES) algorithm [39] with 10 jackknife re-samplings runs. To obtain a stable skeleton map, markers causing unstable neighborhoods and disturbing the monotony of recombination changes were removed. Monotony is a stepwise rf (recombination fraction) increment between a marker and its subsequent neighbors. Then, single markers were added to the map using the ‘extending linkage group’ function with coefficient of enlargement increased stepwise from 1.0 to 1.4. The order of markers was checked for monotony distortion and map size enlargement. Markers, not involved in stable map construction, but corresponded to any defined interval were referred as “attached”. Distribution of markers due to MultiPoint mapping is reported in details in Additional file 1: Table S1 and localization of markers due to N5BT5D/DT5BL analysis and comparison with previously reported 5B mapping data is reported in Additional file 2: Table S2.

Visualization of map was performed by MapChart 2.2 [40]. To compare the obtained map with existing maps we used BioMercator V4.2 software [41, 42].

### QTL analysis

The RICLs were grown in two different conditions in 2014 and 2015. For analysis, genetic linkage map was modified by decreasing the number of markers in order to have distances between markers 2 – 4 cM. For QTL analysis, MultiQTL software [43] was used to perform interval mapping (one trait). To determine QTL threshold LOD value, 1000 permutations were carried out using option “Comparing Hypotheses H1 → H0”. To estimate standard deviations of the main parameters bootstrap analysis was applied. Interval analysis LOD values of heading date trait are presented in Additional file 3: Table S3.

### Determining candidate genes for heading date QTL

To identify the candidate genes for heading date, sequences of SNP markers [44] of the QTL region were analyzed using Gramene BLASTN [45] against *Brachypodium distachyon*, *Oryza sativa* (Indica), *Hordeum vulgare*, *Triticum urartu*, *Triticum aestivum*, *Aegilops tauschii*. Results for obtained sequences from UniProt database [46] were summarized. References to putative proteins are provided. SNPs covered by QTL region and genes, associated with them, are presented in Additional file 4: Table S4 (see also family / domain annotations and implemented description according to InterPro). Then we analyzed probability of these proteins’ involvement in flowering pathways.

## Results

### Sequence analysis of *VRN-B1* genes from CS and CS-5Bdic

The studied lengths of the *VRN-B1* alleles, including promoter and coding regions, from CS and CS-5Bdic were 4780 bp and 4783 bp, respectively. The sequenced region in both cases splits into two parts: 1) the promoter together with exon 1 and the upstream sequence of intron 1; 2) exons 2–8 with the intervening introns. With the exception of a few substitutions located in intron 1, the *VRN-B1* sequence of CS-5Bdic has only one significant change as compared to the CS *VRN-B1* sequence, the insertion of three- nucleotide TCT in exon 7 leading to addition of the amino acid serine (S) in the C- terminal region of the protein. Interestingly, at this position the triplet is tandemly repeated both in CS and CS-5Bdic sequences (6 and 7 times, respectively).

### Analysis of *VRN-1* transcription in CS and CS-5Bdic

**Fig. 1:**
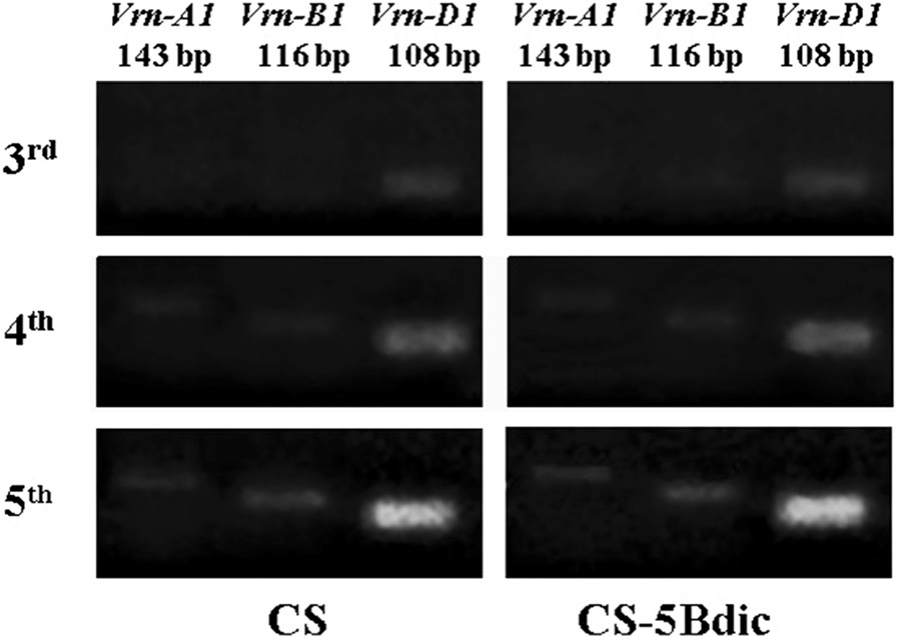
Expression of *VRN-1* homoeoalleles in CS and CS-5Bdic. RT-PCR products were amplified with primers specific for each homoeoallele (see Methods). The length of the PCR products is given above the picture in base pairs. Leaf stages are indicated at the left of the panel

To study *Vrn-1* transcription levels in CS and CS-5Bdic, we performed RT-PCR with RNA samples from leaves of unvernalized plants at different stages before heading: third (3^rd^), fourth (4^th^) and fifth (5^th^) leaf stages. There was no difference between the two lines in the kinetics of transcripts accumulation. The *Vrn-D1* gene is transcribed first and corresponds to the dominant status of this gene in the genome of CS. The transcription level of this gene gradually increased from the 3rd to 5th leaf stages, reaching a maximum at the 5th leaf stage (Fig. 1). Transcripts of two other homoeologous *Vrn-1* genes were detectable starting from the 4th leaf stage with almost no difference from each other in intensity.

### RICLs’ phenotyping and SNP genotyping

**Fig. 2:**
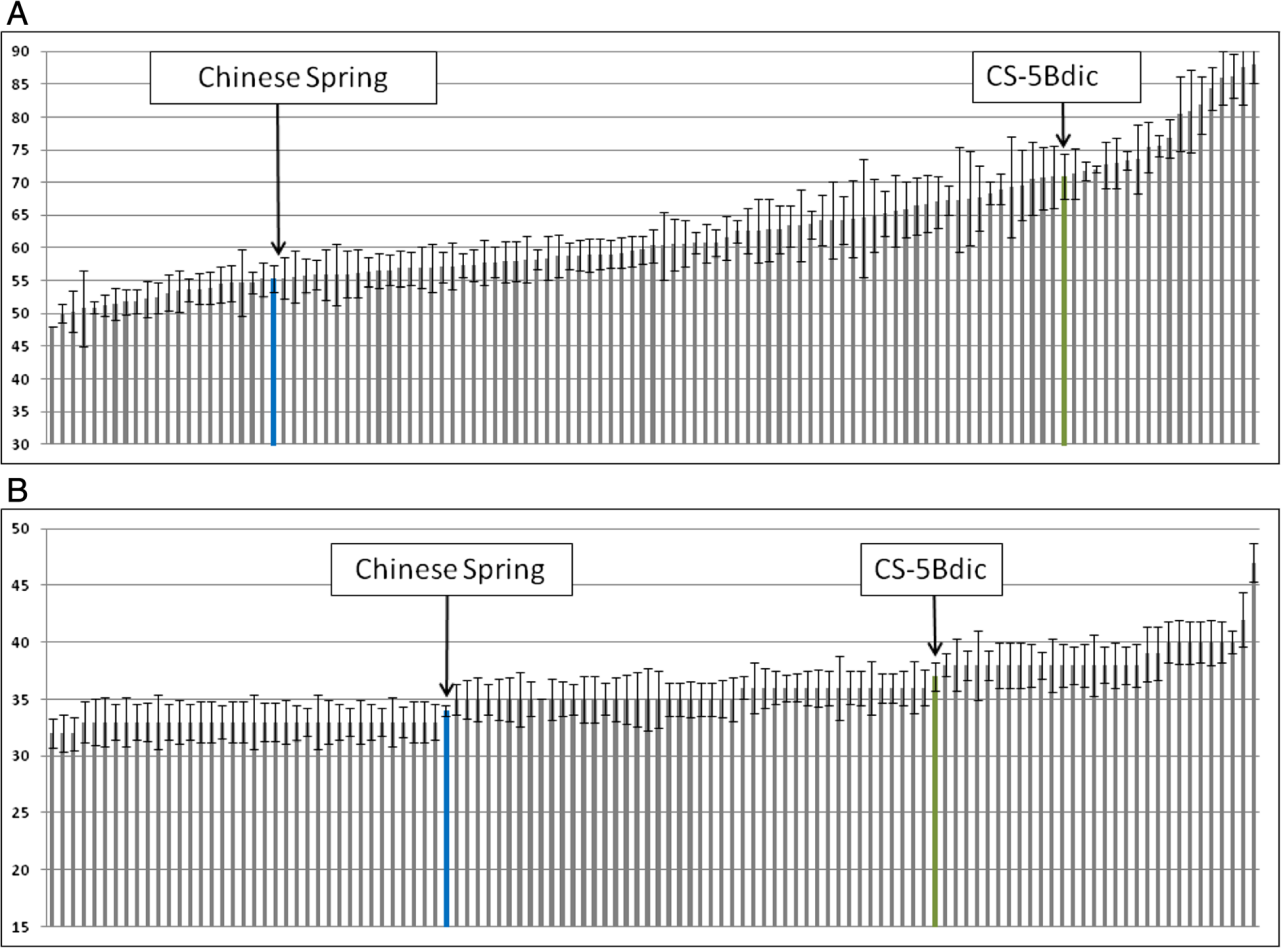
Distribution of heading date of 116 lines with CS and CS-5Bdic (CS is marked in blue color, CS-5Bdic in green). **a** heading date of RICLs without vernalization treatment. **b** heading date of RICLs under the vernalization treatment

As opposed to no differences in expression level of *VRN-1* homoeologous genes in CS and CS-5Bdic, CS-5Bdic was characterized by delayed heading date during the development of recombinant inbred chromosomal lines (RICLs) and the difference in heading date between CS and CS-5Bdic was about 15 days (Fig. 2a).

To identify the loci determining difference in heading time of CS and CS-5Bdic, 116 recombinant inbred chromosomal lines (RICLs) from the cross CS and CS-5Bdic were genotyped using the Illumina Infinium 15 k Wheat platform and phenotyped for several heading date trait. Heading date estimation of vernalized and non-vernalized RICLs was conducted in the controlled conditions of the greenhouse. Heading date variation of non-vernalized RICLs was 48 – 88 days and for the parental lines CS and CS-5Bdic – 55 and 71 days, respectively (Fig. 2a). In the case of vernalized plants, heading date varied between 32 – 47 days and no significant variation was detected (Fig. 2b).

The RICLs were genotyped using 13007 SNP markers of the Illumina Infinium 15 k Wheat platform. Out of these, 418 markers were polymorphic between parental lines CS and CS-5Bdic. Most of the markers proved to be co-dominant (both alleles were detected), while for 10 markers only one parental line was detected. In addition, localization of polymorphic markers on 5BS or 5BL chromosome arm using genotyping data of N5BT5D and DT5BL was also performed.

### Construction of 5B chromosome genetic linkage map

The genetic linkage map was obtained using genotyping data of 116 recombinant inbred lines with 418 polymorphic SNP markers using MultiPoint (version “UltraDense”) software. The length of the resulting map was equal to 80.4 cM (Kosambi mapping function) and consisted of 379 markers in total (with 82 skeletal markers, either of which represented a group of non-recombining (co-segregating) markers). The sizes of such groups varied between 2 to 23 markers. The centromeric region of 5B chromosome was characterized by a high marker density per genetic distance. For instance, there are 11 markers per cM in centromeric region and 2.3 markers per cM in other chromosome regions.

**Fig. 3:**
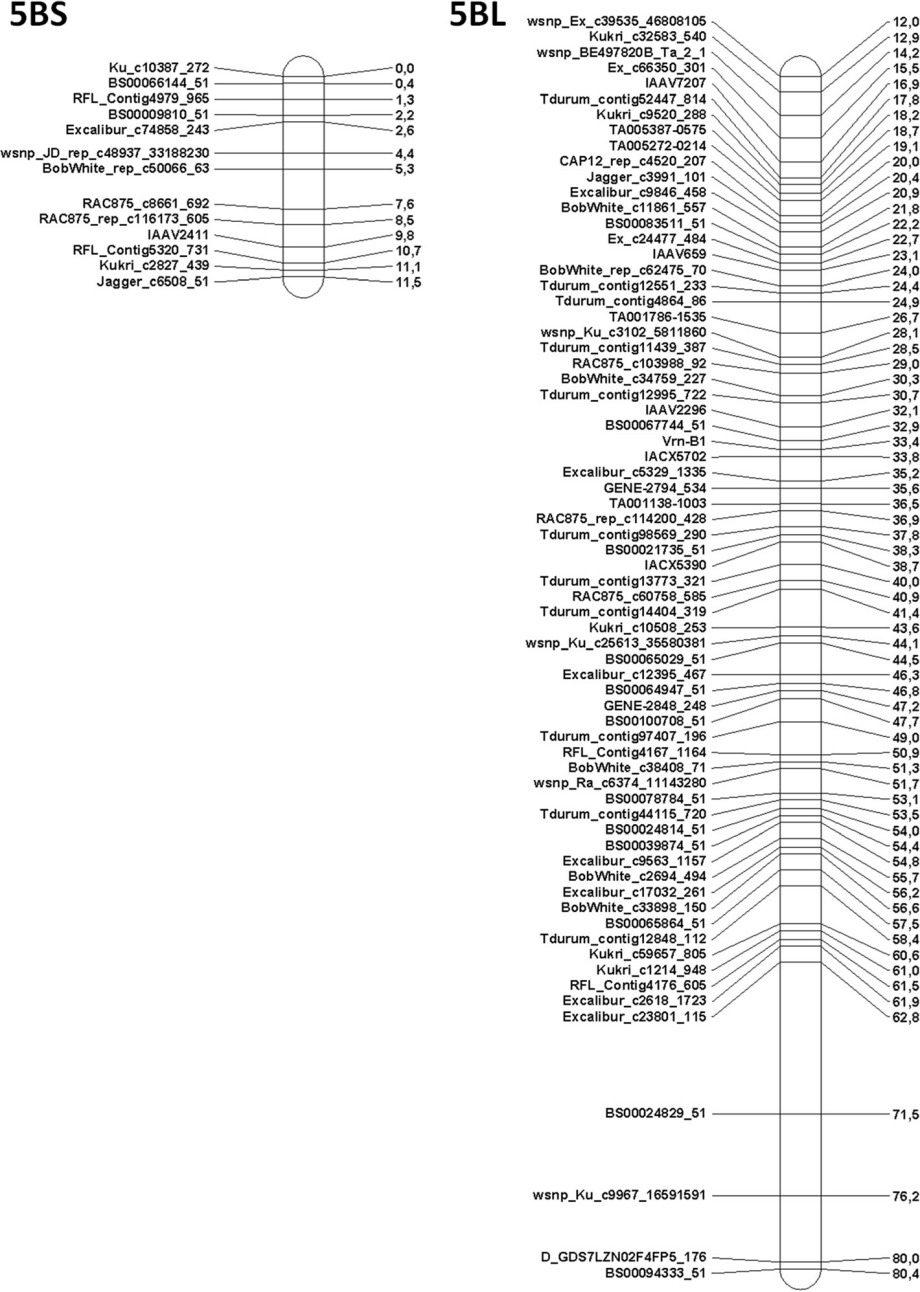
5B chromosome genetic linkage map. This map displays 82 skeletal markers. Co-segregating markers can be found in Additional file 1: Table S1. Distance represented in cM (Kosambi mapping function)

Two markers BS00011514_51 and wsnp_RFL_Contig2809_2587619 were associated with the *VRN-B1* gene and located at 33.4 cM position. These markers including marker to *VRN-B1* formed “bound-together” group (Fig. 3).

### Improvement of SNPs location

To confirm the location of markers on 5BS or 5BL chromosome arms we genotyped nulli-tetrasomic N5BT5D and ditelosomic DT5BL lines and compared data obtained in this investigation with previously represented data. For the first time the following 11 markers were located on 5B chromosome: Tdurum_contig31845_322, Kukri_c32583_540, BS00011102_51, TA005272-0214, RFL_Contig3455_700, BS00065265_51 and TA001999-0466 (chromosome localization of this marker is questionable as it is “attached” marker, not involved in the stable skeletal map), BobWhite_c33898_150, Tdurum_contig12848_112, D_GDS7LZN02F4FP5_176, BS00094333_51 markers. All of these markers were localized on the long arm of 5B chromosome. By reference to SNP genotyping data of RICLs together with N5BT5D and DT5BL lines, the majority of analyzed markers could be exactly assigned either to the short or long arms of 5B chromosome. The centromeric region could be defined between 11.5 cM and 12.0 cM. Comparison with the existing maps enables us to detect multiple localized markers. A summary of data on marker location is presented in Additional file 2: Table S2.

### QTL analysis

During the phenotyping of the material we have noted that significant variation in heading date took place only in non-vernalized lines and parents but not in vernalized plants. Using QTL analysis we detected significant LODs only in experiments with the non-vernalized population. The QTL region was located in the 5BS-5BL pericentromeric region, in the 11.2–18.4 cM interval. The *VRN-B1* gene was localized at 33.4 cM position and no correlation of this locus with heading date was observed in our analysis. The distribution of LOD values is presented in Additional file 3: Table S3.

QTL, associated with heading date, included sets of co-segregating markers. Due to the fact that the SNP markers of the employed array were developed according to coding sequences [27] we are able to compare them with existing databases of model species in order to detect candidate genes underlying heading date variation.

### Identifying candidate genes for heading date variation

The 78 SNPs located in the 11.2–18.4 cM QTL interval were analyzed to identify candidate genes potentially involved in heading date determination. SNP sequences were compared with databases of *Brachypodium distachyon*, *Oryza sativa*, *Hordeum vulgare*, *Aegilops taushii*, *T. urartu* and *T. aestivum* using blastn. We have identified coding sequences overlapping with SNP sequences and detected characteristics of encoded proteins, domains and putative functions. Among the detected candidates there were genes controlling abiotic stress resistance of plants, providing damage protection, phytohormone metabolism, disease resistance and some transcription factors (Additional file 4: Table S4). From this set, we suggest the most probable candidates for heading time control are putative transcription factor genes *WRKY*, *ERF/AP2* and *FHY3*/*FAR1*.

## Discussion

This study was designed to map loci determining heading date difference in two wheat lines, cultivar Chinese Spring and Chinese Spring substitution line with 5B chromosome from *T. dicoccoides*. These lines flowered significantly apart from each other when they were not vernalized. One way to explain these results was to study the known loci associated with the heading date variation, such as *VRN-1*, to analyze the primary sequence and level of transcription in the parental cultivars and the derivative line. The second way is development and study of recombinant inbred chromosomal lines that are different from each other at a single locus or in some loci on 5B chromosome. RICLs are useful genetic models for mapping and trait investigation due to unlimited germplasm for the lines and absence of further segregation in the progeny [47]. Using RILs for constructing high-resolution genetic map is advantageous as the degree of recombination is high and the map positions of tightly linked markers can be determined [48].

### Comparative analysis of the primary structure and expression of the *VRN-B1* gene

Sequencing of the promoter, all exons and 1st intron regions of both *VRN-B1* alleles showed that the allele from CS-5Bdic was almost identical to the allele of CS, except for a few non-significant substitutions in the 1st intron and a three-nucleotide insertion in the coding region. The latter mutation seemed to be most significant since the coding sequences of the *VRN-1* genes, unlike the promoter and 1st intron, are highly conserved even between different species [34, 35]. We did not study the full sequence of the 1st intron because of its size (~9 kb), however, the upstream region of about 2 kb represents the “vernalization critical region” affecting the *VRN-1* expression and response to vernalization [20, 22, 33, 35, 49].

**Fig. 4:**
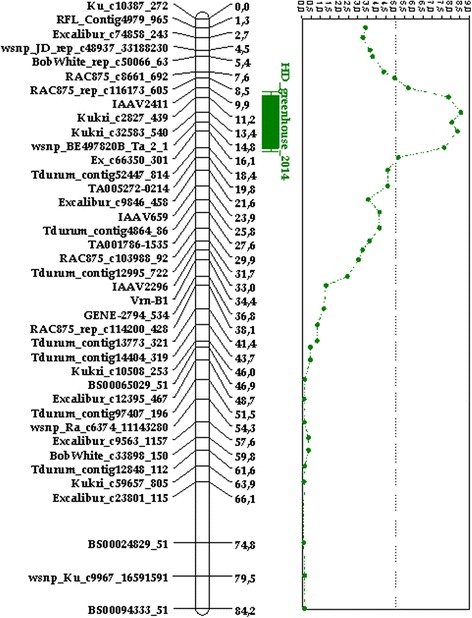
Reduced genetic linkage map with QTL loci heading date

Li et al. [50] showed that one amino acid substitution in the C-terminal region of the VRN-A1 protein leads to sufficient prolongation of vernalization period due to a decreased ability of this protein to bind with the TaHOX1 protein (the first homeobox protein in *T. aestivum*). We found the insertion of a serine in the VRN-B1 amino acid sequence of CS-5Bdic allele, as compared with Vrn-B1 CS and all other Vrn-B1 sequences from databases. As in previous work this insertion also occurred within the C-terminal region. Apparently, the introgressed *VRN-B1* gene from *T. dicoccoides* may participate in protein-protein interactions, determining the growth habit of wheat. However, an effect of this locus on the date of heading has not been established here (Fig. 4). Additional experiments under various conditions of planting are required to clarify whether this mutation is involved in the processes described above.

Another way to influence the vernalization requirements and heading time is modulation of *VRN-1* expression (transcription). Previously, the direct association of the level of *VRN-1* trancription with the heading time of wheat was established [17, 22]. Here, we did not find a difference in the level of transcription of any *VRN-1* loci in CS-5Bdic as compared to CS (Fig. 1). The kinetics of transcription agrees with the previous data showed that in non-vernalized plants the expression of the dominant *Vrn-1* gene starts from the third leaf stage and induces transcription of the recessive homoeoalleles [17, 22]. Consequently, both СS-5Bdic and CS, have only one dominant gene*,* that equally induces the activity of the recessive homoeoalleles *vrn-B1* and *vrn-B1dic*.

### Characterization of genetic linkage map based on CS x CS-5Bdic RICL

In this investigation a map of 5B chromosome was constructed relying on CS x CS-5Bdic using 15 K SNP Illumina wheat platform. There were 52 SNP markers located on the short arm including 13 skeletal markers. The total number of markers on the 5B chromosome genetic linkage map of CS x CS-5Bdic was 379. This corresponded to 5B chromosome maps of tetraploid wheat based on SNP markers [51].

The length of the short arm was 11.54 cM. The total length of 5B long arm was 68.9 including 327 SNP markers with 69 skeletal markers. The resulting map was 80.44 cM. In general, 5B map length for tetraploid wheat corresponded to 206.2 cM [51]. The short length of the 5B map could result from the recombination processes in this certain population (CS x CS-5Bdic). Moreover, some lines (20.7 %) were characterized with the same genotype as parents, where 15.5 % of non-recombinant chromosomes were inherited from CS and 5.2 % from CS-5Bdic. Since CS-5Bdic has alien genetic introgression from the species *T. dicoccoides*, it is likely that alleles of CS parent are more preferable. This data suggest recombination processes between chromosomes from different species to be disturbed.

The centromeric region of 5B chromosome is characterized by high marker density per genetic distance. For instance, there are 11 markers per cM in centromeric region and in distal regions of the chromosome there are 1.1 markers per cM. This is due to low recombination rate in this region and specificity of gene distribution [51]. The earlier studies demonstrated that recombination is suppressed in the pericentromeric region of chromosome 5B, especially in the short arm segment [52, 53].

Skeletal markers were distributed regularly along the chromosome except for the distal part of 5B chromosome long arm (Fig. 3). There were intervals about 4 – 8 cM between Excalibur_c23801_115 and BS00024829_51 (about 8 cM), BS00024829_51 and wsnp_Ku_c9967_16591591 (about 4 cM), Ku_c9967_16591591 and D_GDS7LZN02F4FP5_176 (about 4 cM) skeletal markers, while the average distance between markers was 0.84 cM. However, this region in some maps, reported by Maccaferri et al. [51], revealed the same trend: marker distances were about 4 – 10 cM in the distal part of the 5BL in maps of Meridiano x Claudio, Mohawk x Cocorit and Latino x MG5323 populations. These maps reveal commonality with CS x CS-5Bdic markers in this chromosome region. Perhaps, recombination in this area is not as frequent as in the other parts of the chromosome and there are fewer genes located in this area. Nevertheless, map constructed using genotyping data obtained with this array can be used to compare with more wide-range maps obtained with 90 K Illumina platform.

### Detection of putative determinants of heading date variation

Wheat heading date is strongly influenced by the vernalization genes [5]. Nonetheless QTL (Fig. 4) and expression level (Fig. 1) analyses demonstrated that *VRN-1* is not associated with heading date variation in the non-vernalized CS x CS-5Bdic RICLs.

The other locus on 5B, important for wheat flowering is *Phytochrome C* (*PHY-C*) gene [12]. This gene is associated with *VRN-B1* [11], but no association of these loci with heading time variation in the lines under the investigation was found from the mapping results and QTL analysis.

Zanke et al. [54] have shown marker Kukri_c10016_369 on 5B chromosome to be associated with heading date. This locus is related to the *Hd6* gene family of *Oryza* and has a major impact on heading time in wheat [54]. Kukri_c10016_369 was polymorphic in the RICLs under our investigation and was located in position of 35.6 cM on the map, just below the *VRN-B1* located in 33.4 cM position. However, we didn’t find any significant QTL in this region.

Thus, significant variation of heading date which was observed only in the case of the non-vernalized development and was not associated with the *VRN-B1*, *PHY-C* or *Hd6* genes.

In addition to *VRN-B1*, *PHY-C* and *Hd6* other loci located on 5B chromosome may affect heading date [55]. These loci were shown to be associated with the following markers: *ACA.CTA13/CTCG.CAT7* [56], *Xwmc745–Xcfa21215B.2* [25], *Xgwm371* [24], *wPt-9814 и wPt-4551* [57]. For all of these chromosome regions on 5BL, association with heading date was shown, and most part of them was not associated with the centromere. Although the *eps* locus associated with *Xwmc73* [9] was shown to localize in 5BL centromeric region, but no candidate genes were proposed. Earliness *per se* is the difference in heading time of varieties whose requirements of vernalization and photoperiod have been fulfilled [58] and we observed heading date variation in non-vernalized plants suggesting that this trait is determined by changes in heading time pathway. Moreover, in our investigation heading date QTL were localized in centromeric region of both 5BL and 5BS.

To detect sequences on 5B involved in heading time control we developed a genetic linkage map of the 5B chromosome. Candidate genes for heading date were identified on 5B by comparison with genomes of other species such as purple false brome, rice, barley, common wheat, wild einkorn and goatgrass. The QTL region included some transcription factors, genes involved in stress adaptation, phytochormone metabolism, cytokinesis, intercellular transport, protein folding and photosynthesis.

One of the putative genes influencing heading date is the *WRKY* transcription factor. *WRKY* transcription factors are key regulators of many plant processes, including the responses to biotic and abiotic stresses, senescence, seed dormancy and seed germination, trichome development and epicuticular wax loading, mediating ABA signaling and improvement of the drought tolerance [59–63].

In soybean *WRKY* transcription factors were shown to control flower initiation. *GsWRKY20* positively regulated flowering. *GsWRKY20* was shown to accelerate plant flowering through the regulation of flowering-related genes and floral meristem identity genes [62]. Likewise in Miscanthus *MlWRKY12* TF was suggested to control flowering [64].

However, little is known about the constitution and function of *WRKY* genes in bread wheat (*T. aestivum*) [65]. Common wheat *WRKY* genes are involved in leaf senescing and abiotic stresses [66], plant resistance at the initial stage of cold hardening [67]. WRKY proteins may play important roles in ABA signal transduction pathways involved in both processes of stress tolerance and germination in plants [68]. *TaWRKY* genes were shown to be involved in the abiotic stress response in an ABA-dependent manner [65, 69]. However, no association of *WRKY* genes with flowering has been shown in wheat.

The other candidate gene to modulate heading time is an *ERF/AP2* transcription factor. The *ERF/AP2* family genes were shown to be involved in response to drought, salinity, temperature variation, disease resistance and the flowering control pathway [70, 71]. Arabidopsis *AP2* is the most well characterized gene in the *AP2/ERF* family. *AP2* encodes a putative transcription factor involved in formation of the floral meristem [72], the specification of floral organ identity [73], and the regulation of floral homeotic gene expression [74, 75] in *Arabidopsis* [76].

The other sequence overlapping with SNP in QTL region was a gene involved *FAR-RED ELONGATED HYPOCOTYL3* (*FHY3*) and *FAR-RED-IMPAIRED RESPONSE1* (*FAR1*) domains. *FHY3* and *FAR1* are transcription factors derived from ancient transposases. *FHY3* and *FAR1* were shown to modulate *PhyA*-signaling in higher plants [77] and *PhyA* (*Phytochrome A*) promotes flowering under the long day in Arabidopsis [78, 79]. Other investigations demonstrated that *FHY3* plays a principal role in the circadian clock and heading date control and regulation of heading time by through *ELF4* (*EARLY FLOWERING4*) [77]. Later *FHY3* and *FAR1* were confirmed to integrate light signals into the circadian clock and modulate division of chloroplasts by direct up-regulation of *ELF4* and *ACCUMULATION AND REPLICATION OF CHLOROPLASTS5* (*ARC5*) [77, 80, 81]. In silico analysis of *Oryza sativa* cis-elements suggested that *FAR1/FHY3* is involved in the photoperiod response due to *PHY-A* regulation [82].

The possible reason for heading date delay through introgression from *T. dicoccoides* may be due to the different ancestry of structural genes influencing heading date and transcription factors modulating activity of these genes. Previously, altered gene expression was shown to be common in inter-specific hybrids. For example, reduced expression of introgressed genes was shown in *F3h* [83] and aluminum tolerance genes [84] in wheat-rye hybrids. In wheat lines with barley introgressions a number of genes were shown to be also less expressed [85].

## Conclusion

To study the effect of the introgressed *T. dicoccoides* 5B chromosome on the common wheat *T. aestivum* heading time, we sequenced and analyzed transcription of the most powerful regulator of flowering, *VRN-B1* located on this chromosome. No differences in transcription of different *VRN-1* homeoalleles were observed between CS-5Bdic substitution line and CS. The presence of an additional amino acid in the *VRN-B1* protein sequence of CS-5Bdic implies the possibility of interactions with other proteins taking part in the modulation of flowering and vernalization response.

The set of RICLs, developed in this investigation, provided a good basis for 5B genetic linkage map construction and marker-trait association analysis. Lines were different in their heading date when they were not vernalized. QTL analysis of heading date variation demonstrated that loci in the pericentromeric region of 5B chromosome are significantly associated with heading date. Some genes associated with the SNP markers in this area are likely to be candidates for heading date difference. *WRKY*, *ERF/AP2* and *FHY3*/*FAR1* transcription factors were previously shown to be involved in flowering time modulation. We propose that the probable cause of heading date differences may be due to differences in the origin of interacting heading time pathways and putative transcription factors located on 5B might modulate. Further investigation of the transcription factors and their hypothetic interaction with known heading date genes could help us to further decipher the heading time pathways in cereal crops.

## Additional files

Additional file 1: Table S1. 5B chromosome genetic_linkage_map of CS x CS-5Bdic. The map was constructed using MultiPoint “UltraDense” software. (XLSX 26 kb) Additional file 2: Table S2. Localization of markers due to N5BT5D/DT5BL analysis in current investigation and according to comparison with previously reported 5B mapping data. (XLSX 37 kb) Additional file 3: Table S3. LODs values of Interval analysis of heading date trait. (XLSX 10 kb) Additional file 4: Table S4. Results of blastn analysis for SNP sequences, located in the QTL region. (XLSX 51 kb)

## References

[1] Kitagawa S, Shimada S, Murai K (2012). Effect of Ppd-1 on the expression of flowering-time genes in vegetative and reproductive growth stages of wheat. Genes Genet Syst..

[2] Cockram J, Jones H, Leigh FJ, O’Sullivan D, Powell W (2007). Laurie D a, et al. Control of flowering time in temperate cereals: genes, domestication, and sustainable productivity. J Exp Bot.

[3] Snape JW, Butterworth K, Whitechurch E, Worland AJ (2001). Waiting for fine times: genetics of flowering time in wheat. Euphytica.

[4] Seki M, Chono M, Matsunaka H, Fujita M, Oda S, Kubo K (2011). Distribution of photoperiod-insensitive alleles Ppd-B1a and Ppd-D1a and their effect on heading time in Japanese wheat cultivars. Breed Sci..

[5] Worland AJ (1996). The influence of flowering time genes on environmental adaptability in European wheats Vernalization sensitivity. Euphytica.

[6] Kato K, Yamagata H (1988). Method for evaluation of chilling requirement and narrow-sense earliness of wheat cultivars. Japan J Breed..

[7] Barrett B, Bayram M, Kidwell K (2002). Identifying AFLP and microsatellite markers for vernalization response gene Vrn-B1 in hexaploid wheat using reciprocal mapping populations. Plant Breed.

[8] Leonova I, Pestsova E, Salina E, Efremova T, Roder M, Borner A (2003). Mapping of the Vrn-B1 gene in Triticum aestivum using microsatellite markers. Plant Breed..

[9] Tóth B, Galiba G, Fehér E, Sutka J, Snape JW (2003). Mapping genes affecting flowering time and frost resistance on chromosome 5B of wheat. Theor Appl Genet..

[10] Devos KM, Beales J, Ogihara Y, Doust AN (2005). Comparative sequence analysis of the phytochrome C gene and its upstream region in allohexaploid wheat reveals new data on the evolution of its three constituent genomes. Plant Mol Biol..

[11] Wiebe K, Harris NS, Faris JD, Clarke JM, Knox RE, Taylor GJ (2010). Targeted mapping of Cdu1, a major locus regulating grain cadmium concentration in durum wheat (Triticum turgidum L. var durum). Theor Appl Genet.

[12] Chen A, Li C, Hu W, Lau MY, Lin H, Rockwell NC (2014). Phytochrome C plays a major role in the acceleration of wheat flowering under long-day photoperiod. Proc Natl Acad Sci U S A.

[13] Murai K, Miyamae M, Kato H, Takumi S, Ogihara Y (2003). WAP1, a Wheat APETALA1 Homolog, Plays a Central Role in the Phase Transition from Vegetative to Reproductive Growth. Plant Cell Physiol..

[14] Yan L, Loukoianov A, Tranquilli G, Helguera M, Fahima T, Dubcovsky J (2003). Positional cloning of the wheat vernalization gene VRN1. Proc Natl Acad Sci U S A.

[15] Shitsukawa N, Ikari C, Shimada S, Kitagawa S, Sakamoto K, Saito H (2007). The einkorn wheat (Triticum monococcum) mutant, maintained vegetative phase, is caused by a deletion in the VRN1 gene. Genes Genet Syst..

[16] Trevaskis B, Hemming MN, Dennis ES, Peacock WJ (2007). The molecular basis of vernalization-induced flowering in cereals. Trends Plant Sci..

[17] Loukoianov A, Yan L, Blechl A, Sanchez A, Dubcovsky J (2005). Regulation of VRN-1 vernalization genes in normal and transgenic polyploid wheat. Plant Physiol..

[18] Danyluk J, Kane NA, Breton G, Limin AE, Fowler DB, Sarhan F (2003). TaVRT-1, a putative transcription factor associated with vegetative to reproductive transition in cereals. Plant Physiol..

[19] Trevaskis B, Bagnall DJ, Ellis MH, Peacock WJ, Dennis ES (2003). MADS box genes control vernalization-induced flowering in cereals. Proc Natl Acad Sci U S A..

[20] Von Zitzewitz J, Szucs P, Dubcovsky J, Yan L, Francia E, Pecchioni N (2005). Molecular and structural characterization of barley vernalization genes. Plant Mol Biol..

[21] Trevaskis B, Hemming MN, Peacock WJ, Dennis ES (2006). HvVRN2 Responds to daylength, whereas HvVRN1 is regulated by vernalization and developmental status. Plant Physiol..

[22] Shcherban AB, Khlestkina EK, Efremova TT, Salina EA (2013). The effect of two differentially expressed wheat VRN-B1 alleles on the heading time is associated with structural variation in the first intron. Genetica.

[23] Shindo C, Tsujimoto H, Sasakuma T (2003). Segregation analysis of heading traits in hexaploid wheat utilizing recombinant inbred lines. Heredity.

[24] Hanocq E, Niarquin M, Heumez E, Rousset M, Le Gouis J (2004). Detection and mapping of QTL for earliness components in a bread wheat recombinant inbred lines population. Theor Appl Genet..

[25] Griffiths S, Simmonds J, Leverington M, Wang Y, Fish L, Sayers L (2009). Meta-QTL analysis of the genetic control of ear emergence in elite European winter wheat germplasm. Theor Appl Genet..

[26] Bennett D, Reynolds M, Mullan D, Izanloo A, Kuchel H, Langridge P (2012). Detection of two major grain yield QTL in bread wheat (Triticum aestivum L.) under heat, drought and high yield potential environments. Theor Appl Genet.

[27] Wang S, Wong D, Forrest K, Allen A, Chao S, Huang BE (2014). Characterization of polyploid wheat genomic diversity using a high-density 90 000 single nucleotide polymorphism array. Plant Biotechnol J..

[28] Avni R, Nave M, Eilam T, Sela H, Alekperov C, Peleg Z (2014). Ultra-dense genetic map of durum wheat × wild emmer wheat developed using the 90 K iSelect SNP genotyping assay. Mol Breed..

[29] GrainGenes: A Database for Triticeae and Avena. http://wheat.pw.usda.gov/GG3/map_summary. Accessed 5 May 2015.

[30] Akhunov E, Nicolet C, Dvorak J (2009). Single nucleotide polymorphism genotyping in polyploid wheat with the Illumina GoldenGate assay. Theor Appl Genet..

[31] Zhao K, Aranzana MJ, Kim S, Lister C, Shindo C, Tang C (2007). An Arabidopsis example of association mapping in structured samples. PLoS Genet..

[32] Aranzana MJ, Kim S, Zhao K, Bakker E, Horton M, Jakob K (2005). Genome-wide association mapping in Arabidopsis identifies previously known flowering time and pathogen resistance genes. PLoS Genet..

[33] Shcherban AB, Efremova TT, Salina EA (2012). Identification of a new Vrn-B1 allele using two near-isogenic wheat lines with difference in heading time. Mol Breed..

[34] Yan L, Helguera M, Kato K, Fukuyama S, Sherman J, Dubcovsky J (2004). Allelic variation at the VRN-1 promoter region in polyploid wheat. Theor Appl Genet..

[35] Fu D, Szucs P, Yan L, Helguera M, Skinner JS, von Zitzewitz J (2005). Large deletions within the first intron in VRN-1 are associated with spring growth habit in barley and wheat. Mol Genet Genomics..

[36] Himi E, Nisar A, Noda K (2005). Colour genes (R and Rc) for grain and coleoptile upregulate flavonoid biosynthesis genes in wheat. Genome..

[37] TraitGenetics GmbH. http://www.traitgenetics.com/en/. Accessed 1 May 2015.

[38] Ronin Y, Minkov D, Mester D, Akhunov E (2013). Building ultra-dense genetic maps in the presence of genotyping errors and missing data.

[39] Ronin Y, Mester D, Minkov D, Korol A (2010). Building reliable genetic maps: different mapping strategies may result in different maps. Nat Sci..

[40] Voorrips RE (2002). MapChart: Software for the Graphical Presentation of Linkage Maps and QTLs. J. Hered..

[41] Sosnowski O, Charcosset A, Joets J (2012). Biomercator V3: An upgrade of genetic map compilation and quantitative trait loci meta-analysis algorithms. Bioinformatics.

[42] BioMercator V3: a software for genetic map compilation, QTL meta-analyses. http://moulon.inra.fr/biomercator. Accessed 29 May 2015.

[43] Korol A, Mester D, Frenkel Z, Ronin Y, Feuillet C, Muehlbauer GJ (2009). Methods for Genetic Analysis in the Triticeae. Genetics and Genomics of the Triticeae.

[44] The Triticeae Toolbox (T3) Available from: https://triticeaetoolbox.org/wheat/. Accessed 10 July 2015.

[45] Gramene: A comparative resource for plants (Release 46) Available from: http://ensembl.gramene.org/Tools/Blast?db=core. Accessed 12 July 2015.

[46] The Universal Protein Resource (UniProt). http://www.uniprot.org/. Accessed 21 July 2015.

[47] Singh BD, Singh AK (2015). Mapping Populations. Marker-Assisted Plant Breeding: Principles and Practices.

[48] Boopathi NM (2013). Genetic Mapping and Marker Assisted Selection.

[49] Dubcovsky J, Loukoianov A, Fu D, Valarik M, Sanchez A, Yan L (2006). Effect of photoperiod on the regulation of wheat vernalization genes VRN1 and VRN2. Plant Mol Biol..

[50] Li G, Yu M, Fang T, Cao S, Carver BF, Yan L (2013). Vernalization requirement duration in winter wheat is controlled by Ta VRN-A1 at the protein level. Plant J..

[51] Maccaferri M, Ricci A, Salvi S, Milner SG, Noli E, Martelli PL (2014). A high-density, SNP-based consensus map of tetraploid wheat as a bridge to integrate durum and bread wheat genomics and breeding. Plant Biotechnol J..

[52] Sourdille P, Singh S, Cadalen T, Brown-Guedira GL, Gay G, Qi L (2004). Microsatellite-based deletion bin system for the establishment of genetic-physical map relationships in wheat (Triticum aestivum L.). Funct Integr Genomics.

[53] Timonova EM, Dobrovol’skaya OB, Sergeeva EM, Bildanova LL, Sourdille P, Feuillet C (2013). A comparative genetic and cytogenetic mapping of wheat chromosome 5B using introgression lines. Russ J Genet..

[54] Zanke C, Ling J, Plieske J, Kollers S, Ebmeyer E, Korzun V (2014). Genetic architecture of main effect QTL for heading date in European winter wheat. Front Plant Sci..

[55] Milec Z, Valárik M, Bartoš J, Safář J (2014). Can a late bloomer become an early bird? Tools for flowering time adjustment. Biotechnol Adv..

[56] Marza F, Bai GH, Carver BF, Zhou WC (2006). Quantitative trait loci for yield and related traits in the wheat population Ning7840 x Clark. Theor Appl Genet..

[57] Le Gouis J, Bordes J, Ravel C, Heumez E, Faure S, Praud S (2012). Genome-wide association analysis to identify chromosomal regions determining components of earliness in wheat. Theor Appl Genet..

[58] Kato K, Wada T (1999). Genetic analysis and selection experiment for narrow-sense earliness in wheat by using segregating hybrid progenies. Breed Sci..

[59] Rushton DL, Tripathi P, Rabara RC, Lin J, Ringler P, Boken AK (2012). WRKY transcription factors: Key components in abscisic acid signalling. Plant Biotechnol J..

[60] Johnson CS, Kolevski B, Smyth DR (2002). TRANSPARENT TESTA GLABRA2, a trichome and seed coat development gene of Arabidopsis, encodes a WRKY transcription factor. Plant Cell..

[61] Wang H, Hao J, Chen X, Hao Z, Wang X, Lou Y (2007). Overexpression of rice WRKY89 enhances ultraviolet B tolerance and disease resistance in rice plants. Plant Mol Biol..

[62] Luo X, Sun X, Liu B, Zhu D, Bai X, Cai H (2013). Ectopic Expression of a WRKY Homolog from Glycine soja Alters flowering time in Arabidopsis. PLoS One.

[63] Luo X, Xi B, Sun X, Zhu D, Liu B, Ji W (2013). Expression of wild soybean WRKY20 in Arabidopsis enhances drought toleranc. J Exp Bot.

[64] Yu Y, Hu R, Wang H, Cao Y, He G, Fu C (2013). MlWRKY12, a novel Miscanthus transcription factor, participates in pith secondary cell wall formation and promotes flowering. Plant Sci..

[65] Zhu X, Liu S, Meng C, Qin L, Kong L, Xia G (2013). WRKY transcription factors in wheat and their induction by biotic and abiotic stress. Plant Mol Biol Report..

[66] Li W, Lin Z, Zhang X (2007). A Novel segregation distortion in intraspecific population of asian cotton (Gossypium arboretum L.) detected by molecular markers. J Genet Genomics.

[67] Talanova VV, Titov AF, Topchieva LV, Malysheva IE, Venzhik YV, Frolova SA (2009). Expression of WRKY transcription factor and stress protein genes in wheat plants during cold hardening and ABA treatment. Russ J Plant Physiol..

[68] Xu Q, Feng WJ, Peng HR, Ni ZF, Sun QX (2014). TaWRKY71, a wrky transcription factor from wheat, enhances tolerance to abiotic stress in transgenic Arabidopsis thaliana. Cereal Res Commun..

[69] WANG R, WU H-L, ZHANG M, Zhong-Fu N, SUN Q-X (2013). Cloning, characterization and transgenic function analysis of wheat (Triticum aestivum L.) TaWRKY51. Gene J Agric Biotechnol..

[70] Yamaguchi-Shinozaki K, Shinozaki K (2006). Transcriptional regulatory networks in cellular responses and tolerance to dehydration and cold stresses. Annu Rev Plant Biol..

[71] Zhuang J, Peng RH, Cheng ZM, Zhang J, Cai B, Zhang Z (2009). Genome-wide analysis of the putative AP2/ERF family genes in Vitis vinifera. Sci Hortic..

[72] Irish VF, Sussex IM (1990). Function of the apetala-1 gene during Arabidopsis floral development. Plant Cell..

[73] Kunst L, Klenz JE, Martinez-Zapater J, Haughn GW (1989). AP2 Gene Determines the Identity of Perianth Organs in Flowers of Arabidopsis thaliana. Plant Cell..

[74] Drews GN, Bowman JL, Meyerowitz EM (1991). Negative regulation of the Arabidopsis homeotic gene AGAMOUS by the APETALA2 product. Cell..

[75] Mandel MA, Bowman JL, Kempin SA, Yanofsky MF (1992). Manipulation of flower structure in transgenic tobacco. Cell..

[76] Kim S, Soltis PS, Wall K, Soltis DE (2006). Phylogeny and domain evolution in the APETALA2-like gene family. Mol Biol Evol..

[77] Li G, Siddiqui H, Teng Y, Lin R, Wan X, Li J (2011). Coordinated transcriptional regulation underlying the circadian clock in Arabidopsis. Nat. Cell Biol. [Internet].

[78] Johnson E, Bradley M, Harberd NP, Whitelam CC (1994). Photoresponses of light-crown phya mutants of Arabidopsis. Plant Physiol..

[79] Neff MM, Chory J (1998). Genetic interactions between phytochrome A, phytochrome B, and cryptochrome 1 during Arabidopsis development. Plant Physiol..

[80] Limin AE, Fowler DB (2006). Low-temperature tolerance and genetic potential in wheat (Triticum aestivum L.): Response to photoperiod, vernalization, and plant development. Planta.

[81] Ouyang X, Li J, Li G, Li B, Chen B, Shen H (2011). genome-wide binding site analysis of far-red elongated hypocotyl3 reveals its novel function in arabidopsis development. Plant Cell..

[82] Mongkolsiriwatana C, Pongtongkam P, Peyachoknagul S (2009). In silico promoter analysis of photoperiod-responsive genes identified by DNA microarray in rice (Oryza sativa L.). J Nat Sci.

[83] Khlestkina EK, Tereshchenko OY, Salina EA (2009). Anthocyanin biosynthesis genes location and expression in wheat-rye hybrids. Mol Genet Genomics.

[84] Gustafson JP, Ross K (1990). Control of alien gene expression for aluminum tolerance in wheat. Genome..

[85] Taketa S, Takeda K (1997). Expression of dominant marker genes of barley in wheat-barley hybrids. Genes Genet Syst..

